# Factors affecting the outcomes of direct pulp capping using Biodentine

**DOI:** 10.1007/s00784-017-2296-7

**Published:** 2017-12-12

**Authors:** Mariusz Lipski, Alicja Nowicka, Katarzyna Kot, Lidia Postek-Stefańska, Iwona Wysoczańska-Jankowicz, Lech Borkowski, Paweł Andersz, Anna Jarząbek, Katarzyna Grocholewicz, Ewa Sobolewska, Krzysztof Woźniak, Agnieszka Droździk

**Affiliations:** 1 Department of Preclinical Conservative Dentistry and Preclinical Endodontics, Pomeranian Medical University of Szczecin, Al. Powstańców Wlkp. 72, 70-111 Szczecin, Poland; 2 Department of Conservative Dentistry and Endodontics, Pomeranian Medical University of Szczecin, Szczecin, Poland; 3 Department of Pediatric Dentistry, Silesian Medical University, Zabrze, Poland; 4 Private Practice, Szczecin, Poland; 5 Department of General Dentistry, Pomeranian Medical University of Szczecin, Szczecin, Poland; 6 Department of Gerodontology, Pomeranian Medical University of Szczecin, Szczecin, Poland; 7 Department of Orthodontics, Pomeranian Medical University of Szczecin, Szczecin, Poland

**Keywords:** Biodentine, Carious pulp exposure, Direct pulp capping, Treatment outcome

## Abstract

**Introduction:**

This study aimed to evaluate the prognostic value of factors with regard to the treatment outcome of direct pulp capping using Biodentine (Septodont, Saint-Maur-des-Fossés, France), in permanent teeth in which the pulps were exposed during caries removal.

**Methods:**

Between 2010 and 2014, 112 teeth with deep carious lesions underwent direct pulp capping. The patients were followed up at 2–3 months and 1–1.5 years with a routine examination on both recall visits. Periapical radiographs were taken at 1–1.5 years. Lack of patient complaints, positive reactions to cold and electric testing, no sensitivity to percussion, and no widening of the periapical ligament indicated success. The Fisher exact test was used for statistical analysis. The significance level was *P* = .05.

**Results:**

Eighty-six teeth were available for 1–1.5 years follow-up. The overall success rate was 82.6%. Only age had a significant effect on the pulpal survival rate: the success rate was 90.9% in patients younger than 40 years and 73.8% in patients 40 years or older (*P* = .0480). Sex, initial or secondary caries treatment, occlusal or cervical/proximal caries, delayed placement of permanent filling, tooth position, and arch type did not influence the outcome.

**Conclusions:**

A patient’s age influenced the outcome of direct pulp capping using this new calcium silicate cement.

**Clinical relevance:**

Asymptomatic vital permanent teeth with cariously exposed pulp can be treated successfully by direct pulp capping using Biodentine.

Direct pulp capping is a procedure in which a medicament, dressing, or dental material is placed directly over exposed dental pulp to preserve its vitality. Inducing reparative tertiary dentin formation by pulp cells has been widely accepted as the ultimate goal of using capping material [1, 2]. For many decades, calcium hydroxide was the material of choice among the various available pulp-capping agents [1, 2]. However, there are shortcomings when using this material such as its dissolution in tissue fluids and degradation on tooth flexure, the formation of tunnel defects beneath dentinal bridges, and poor sealing [3–6].

An alternative gold standard, mineral trioxide aggregate (MTA), is available as a direct pulp-capping material [7–9]. However, MTA is difficult to use because of its long setting time, poor handling properties, cost, and the potential discoloration of teeth and soft tissue [10–12]. To overcome some of these limitations, other bioactive tricalcium silicate cements have been recently introduced on the market. One material is Biodentine. It consists of a powder and liquid. The powder primarily contains tricalcium silicate (3CaO·SiO_2_) and dicalcium silicate (2CaO·SiO_2_) and calcium carbonate (CaCO_3_). Zirconium dioxide (ZrO_2_) is a contrast medium. The liquid consists of calcium chloride (CaCl_2_·2H_2_O), which is used as a setting accelerator and water-reducing agent in aqueous solution with an admixture of polycarboxylate (i.e., a superplasting agent). Mixing is achieved by using an amalgamator for 30 s at 4000–4200 rpm. The manufacturer has specified the powder to liquid ratio. This allows practitioners to achieve a reproducible material with optimum properties each time. The initial setting time according to the manufacturer is about 12 min. However, Kaup et al. [13] evaluated the final setting time of this material to be 85 min according to ISO 6876:2001. The consistency of Biodentine is similar to that of phosphoric cement [13, 14].

Several in vitro studies have evaluated Biodentine [14–17]; however, few histologic studies have been conducted to evaluate the pulp response of this calcium silicate cement in animal teeth [18–20] and in human teeth [21, 22]. A survey of the available literature shows that a few isolated clinical investigations have been published that include the use of Biodentine for direct pulp capping in permanent teeth, but no studies have an adequate sample size or long-term data [23–26].

This study aimed to evaluate the prognostic value of factors with regard to treatment outcome of direct pulp capping using the new calcium silicate cement Biodentine in permanent teeth in which the pulp was exposed during caries removal. The tested null hypothesis was that the following factors would not influence the results of direct pulp capping using Biodentine: (1) sex, (2) age of < 40 years or ≥ 40 years, (3) anterior or posterior tooth, (4) maxilla or mandible, (5) initial or secondary caries treatment, (6) occlusal or proximal/cervical caries localization, and (7) immediate restoration (> 1 day) or delayed placement of a permanent filling (2–3 months).

## Material and methods

All patients in this study were referred for routine conservative treatment at the Department of Pediatric Dentistry of Pomeranian Medical University of Szczecin (Szczecin, Poland), the Department of Preclinical Conservative Dentistry and Preclinical Endodontics of Pomeranian Medical University of Szczecin (Szczecin, Poland), the Department of Pediatric Dentistry of Silesian Medical University (Zabrze, Poland), or private dental offices (in Szczecin, Poland) between 2010 and 2014. The treatment was performed by a total of six dentists who had limited their work to conservative dentistry for 10–25 years. Each dentist received training in the use of Biodentine for direct pulp capping to ensure uniformity among the operators.

This study comprised 112 vital teeth with caries-induced pulp exposure, which were obtained from 94 patients (50 female patients and 44 male patients, age 11–79 years). The patients and/or their legal guardians received thorough explanations concerning the clinical procedures and possible complications. All procedures were registered by the local ethical committee of the Pomeranian Medical University (Szczecin, Poland; KB-0012/96/11/15).

Direct pulp capping was indicated when a tooth was exposed on account of caries excavation. However, during the preparation, efforts were made not to expose the pulp. In these patients, the appropriate materials were used for indirect capping. Only teeth with signs and/or symptoms of reversible pulpitis were included. Teeth exhibiting reversible pulpitis had provoked pain of short duration that was relieved on the removal of the stimulus (i.e., cold, heat, or compressed air). Teeth were excluded that exhibited signs and/or symptoms of irreversible pulpitis such as prolonged unbearable pain or pain disturbing night sleep, lack of response to cold (Kȁltespray, M&W Dental GmbH, Büdingen, Germany; Aethylum Chloratum, Filofarm, Bydgoszcz, Poland) and electrical pulp testing (Vitality Scanner Pulp Vitality Tester, SybronEndo, Orange, CA or Gentle Pulse Analog Pulp Tester, Parkell, Edgewood, NY), a sinus tract or swelling, percussion pain, periodontal inflammation, radiographic evidence of calcification of the pulp chamber or canals, internal/external resorption, or furcal/periapical radiolucency. Furthermore, no treated patient took corticosteroids or statins or was pregnant before or during this study.

After local anesthesia, teeth were isolated and disinfected with 0.2% chlorhexidine before caries removal. Round diamond burs and high-speed handpiece with copious water spray were used to remove enamel. Rose burs and a low-speed handpiece under water/air spray coolant were used to remove carious dentin. When the pulp was exposed by the caries excavation process, the cavity was rinsed with sterile saline and a cotton pellet soaked with 2% sodium hypochlorite, 2% chlorhexidine, or sterile saline, which was left at the pulpal wall of the cavity. Teeth with excessive uncontrollable bleeding were excluded from the study. After controlling the bleeding, Biodentine was mixed according to the manufacturer’s instructions and applied on the exposed pulp. Cavities were restored directly without provisional filling (i.e., one-stage treatment) or restored provisionally (i.e., two-stage treatment). The decision depended on the patient’s time availability.

For the one-stage group, Biodentine was placed with a flat hand instrument over the exposed pulp and surrounding dentin and then lightly condensed with a ball burnisher to achieve a thickness of 2–3 mm. After 12–20 min of Biodentine hardening, a glass ionomer cement was used as the base (Ketac Bond, 3M ESPE, Seefeld, Germany; Fuji Triage, GC Corporation, Tokyo, Japan; ChemaDent G-J-P NR 3, Chema-Elektromet Rzeszów, Poland; and Vitrebond, 3M ESPE, Seefeld, Germany). The cavities were restored with a light-cured resin composite (Herculite XRV, Kerr, Bioggio, Switzerland; Gradia, GC Corporation, Tokyo, Japan; Charisma, Heraeus Kulzer Inc., Armonk, NY; N’Durance, Septodont, Saint-Maur-des-Fossés, France; Synergy D6, Coltène/Whaledent Inc., Cuyahoga Falls, OH; and Spectrum, Dentsply Caulk, Milford, DE). All materials were applied in accordance with the manufacturer’s directions with dedicated bonding systems. Prelude (Danville, Carlsbad, CA) was used with composite materials which had no their own bonding system.

For class II cavities, a thin metallic matrix was carefully wedged with wooden wedges. After checking the occlusion/articulation, the final finishing was performed. For the two-stage treatment group, Biodentine was applied for direct pulp capping and for temporary restorations. For class II cavities, a metallic matrix was also used. After hardening the material (12–20 min), the occlusion was gently checked. A carving instrument was used for occlusal adjustment. At the subsequent visit (2–3 months after capping), the Biodentine material was partially removed with round diamond burs on a high-speed handpiece with copious water spray to maintain a minimum cement thickness of 2–3 mm to protect the pulp, and restored with a resin composite filling (i.e., the same process as for the one-stage treatment).

## Follow-up examination

The patients were followed up at 2–3 months and 1–1.5 years. All patients had a routine examination during both recall visits. A periapical radiograph was taken at 1–1.5 years. One patient had cone-beam computed tomography but no periapical radiographs. Two blinded observers independently examined the radiographs and reached a consensus for all teeth. Periapical pathology was diagnosed if the apical part of the periodontal ligament was at least twice as wide as in other parts of the roots and the lamina dura was absent. In addition, the pulpal space was checked for calcific alterations in comparison with the adjacent teeth, which had not been pulp-capped. The teeth that exhibited pulpal vitality and did not show any clinical or radiographic signs and/or symptoms of irreversible pulpitis and pulp necrosis/apical periodontitis were considered a treatment success. The teeth with no response to the pulp vitality test and teeth that exhibited clinical or radiographic signs and/or symptoms of irreversible pulpitis or necrosis/apical periodontitis were considered treatment failures. The assessment included tooth crown discoloration. The color of the treated tooth and the adjacent teeth was compared during visual examination. If the patient was not examined at these intervals, a telephone interview was attempted. Patients who dropped out of the study refused to participate in the recall.

## Statistical analysis

The Fisher exact test was used for analysis with a significance level of *P* < .05.

## Results

Among the 112 teeth included in this study, 91 teeth (81.2%) were examined at 2–3 months and 86 teeth (76.4%) at 1–1.5 years (the median follow-up period was 14.7 months).

At the time of treatment with direct pulp capping, the patients’ age ranged 11–79 years with a median age of 44 years. The overall success rates for the 2–3-month and 1–1.5-year recall groups were 100 and 82.6%, respectively. Of the 86 teeth recalled at 1–1.5 years, the outcome was deemed as a failure in 15 (17.4%) teeth. Nine of the failed teeth received subsequent root canal treatment, five teeth showed evidence of pulp necrosis at follow-up, and one tooth (a third molar) was extracted. The pulp space in five teeth was substantially obliterated, but the teeth were responsive to vitality testing and did not show any signs of clinical or radiographic failures (i.e., no complaints of pain or tenderness to percussion or palpation, no evidence of periradicular bone or external/internal root resorption on radiographs); therefore, the outcome was considered a success in these teeth.

The outcome was not influenced by the following parameters: sex, initial or secondary caries treatment, occlusal or cervical caries localization, delayed placement of permanent filling, tooth position, and arch type. Thus, the null hypothesis was accepted for these factors.

**Fig. 1:**
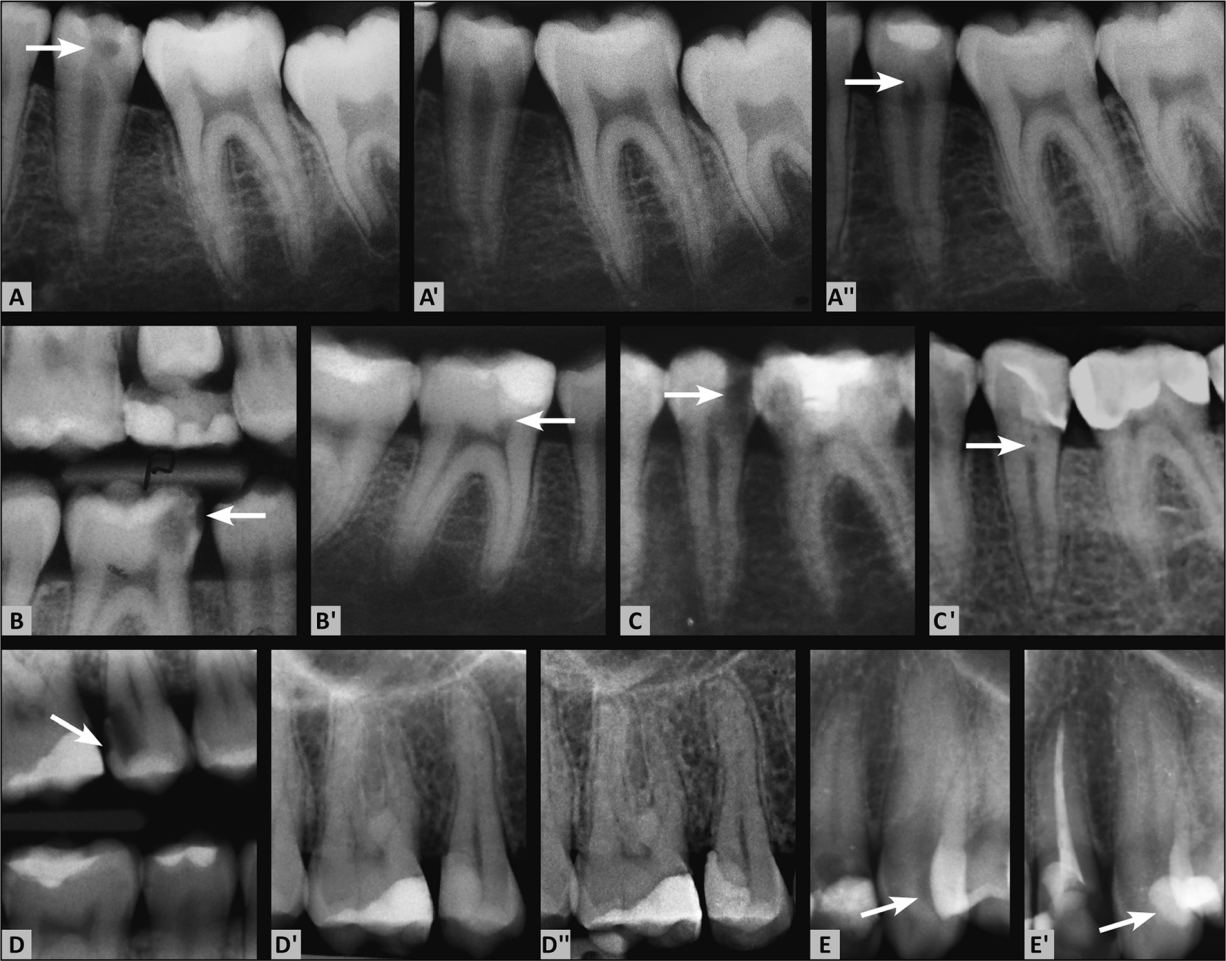
(A) The preoperative periapical radiograph shows a deep carious lesion in the second mandibular premolar (arrow). (A′) Postoperative radiograph taken directly after the application of Biodentine for pulp capping and for temporary restoration. (A″) Follow-up radiograph after 18 months. There is dentine bridge formation and the development of the tooth (arrow). (B) The bitewing radiograph shows a deep carious lesion on the first mandibular molar (arrow). (B′) The periapical radiograph after 18 months after direct capping does not show any pathological findings on tooth 46. There is dentine bridge formation (arrow). (C) The preoperative radiograph of the mandibular premolar shows a deep carious lesion (arrow). (C′) The follow-up radiograph at 12 months shows dentine bridge formation (arrow). (D) The preoperative bitewing radiograph shows a deep carious lesion in the second maxillary premolar (arrow). (D′) The postoperative radiograph taken directly after the application of Biodentine for pulp capping and for temporary restoration. (D″) The follow-up radiograph at 14 months. (E) The preoperative periapical radiograph of the maxillary canine shows a deep carious lesion on the distal surface (arrow). (E′) The periapical radiograph at 12 months after direct capping does not show any pathological findings of this tooth

However, the patients’ age did influence the outcome; therefore, the null hypothesis concerning this parameter was rejected (Table 1). Figure 1 shows examples of the treated teeth.

**Table 1:** Outcome of direct pulp capping with Biodentine with regard to the study variables

Studied variables	Success		Failure		*P* value
	*n*	%	*n*	%	
Age					
< 40 years	40	90.9	4	9.1	.0480
≥ 40 years	31	73.8	11	26.2	
Sex					
Female	40	83.3	8	16.7	1
Male	31	81.6	7	18.4	
Tooth location					
Maxilla	34	87.2	5	12.8	.3963
Mandible	37	78.7	10	21.3	
Tooth type					
Anterior	10	76.9	3	23.1	.6911
Posterior	61	83.6	12	16.4	
Caries treatment					
Initial	39	88.6	5	11.4	.1608
Secondary	32	76.2	10	23.8	
Caries location					
Occlusal	19	95.0	1	5.0	.1749
Proximal/cervical	52	78.8	14	21.2	
Time before permanent restoration					
Direct	42	85.7	7	14.3	.4031
2–3 months	29	78.4	8	21.6

The follow-up visit at 2–3 months showed no obvious crown discoloration of the treated teeth. However, 6 of 71 teeth with vital pulp seemed to be yellower than the adjacent teeth after 1–1.5 years. There was no gray discoloration of the teeth.

## Discussion

This study aimed to identify the significant prognostic factors of treatment success in direct pulp capping using a new calcium silicate-based cement, Biodentine. Thus, no control groups using calcium hydroxide and/or MTA were included. Our study investigated similar prognostic factors that were used in previous clinical studies that assessed the effectiveness of calcium hydroxide and/or MTA [27–34]. Several factors were investigated: sex, age, primary or secondary caries, occlusal or proximal/cervical caries, delayed placement of permanent filling, size of exposure, tooth position, and arch type. However, the outcomes indicated that only age had a significant effect on the survival rate of vital pulps.

## Age

Patient age may have a role in the survival rate after direct pulp capping [27–33]. In the present study, the age-dependent success of treatment was statistically significant (*P* = .048). A success rate of 90.9% occurred in patients younger than 40 years and 73.8% in patients 40 years and older. This finding is in accordance with that of Cho et al. [27], who reported that age had significant effects on the survival rate: patients younger than 40 years had a better success rate than older patients. Dammaschke et al. [30] reported significantly lower favorable treatment outcomes for direct pulp capping in the oldest age cohort (i.e., > 60 years), compared to patients younger than 40 years old. Hørsted et al. [31] found significant differences between the youngest patients (i.e., 10–29 years) and the oldest patients (i.e., 50–79 years). However, some studies could not confirm an influence of age on the success or failure of pulp-capped teeth [29, 35].

The higher success rate for patients younger than 40 years than for patients 40 years old and older can be explained by the high capacity of pulp tissue in young patients. Indirect comparison of the weighted pooled success rate in teeth with an open apex (i.e., higher healing capacity of the pulp tissue) or closed apex showed statistically more successful outcomes in teeth with incomplete root development [36]. However, further clinical studies with a higher number of patients are needed to re-evaluate the age-related influence on treatment outcome after pulp capping with Biodentine.

## Sex

Available studies show a patient’s sex has no significant effect on the treatment outcome of direct pulp capping [27–35]. In the present study, the success rates, as expected, also did not differ between female and male patients.

## Tooth type and location

The type of tooth (anterior vs. posterior) and location (mandible vs. maxillary arch) showed no significant difference in the survival rate. This finding is in accordance with the findings of various studies [27, 30, 32, 34]. Some authors state that the anterior teeth had a higher failure rate of treatment outcome than the posterior teeth [31, 37]. However, other studies showed more favorable treatment outcome in the anterior teeth than in the posterior teeth [38].

## Primary caries versus secondary caries

In the present study, teeth with primary caries demonstrated a higher success rate (88.6%), compared to teeth with secondary caries (76.2%). However, no statistically significant difference existed (*P* = .1608). The influence of this factor on the success of direct pulp-capping treatment was studied only by Marques et al. [33], who used MTA and reported 88.9% treatment success in teeth with secondary caries, compared to 94.7% treatment success in teeth with primary caries. However, in the cited study, the difference was also not statistically significant.

## Caries location

The exposure site (occlusal vs. cervical/proximal) could affect the survival rate. Cho et al. [27] reported a better survival rate when the exposure site was limited to the occlusal side than when it was on the axial side. Treatment failure occurred because of difficulty of isolating axial exposure from contaminants rather than because of pulp exposure on the occlusal side, completing caries removal, applying the pulp-capping material, or sealing the cavity. Jang et al. [34] observed that two thirds of treatment failures involved restorations of teeth with class V cavities caused by root caries; the failure rate was 50%, which was the highest rate, compared to the groups with class I, II, or III cavities. The sealing quality of a class V restoration may be reduced because of insufficient cavity volume for capping material and permanent restoration and tensile stress under occlusal loading. In the present study, no statistically significant difference was evident between occlusal and cervical/proximal exposure. This confirms the findings of a study by Pereira and Stanley [39], who did not detect any difference for treatment success based on the site of exposure.

## Time span before permanent restoration

Some research shows that the time span before the placement of a permanent restoration after pulp capping has a major impact on the healing of an exposed pulp; a permanent restoration protects the tooth structures more effectively from microleakage, compared to a temporary filling [28]. Barthel et al. [32] reported a significantly higher failure rate in teeth with a temporary restoration, compared to a permanent amalgam, composite, or gold cast restoration.

Multivariate analyses in another study [29] confirmed an increased risk of failure after pulp capping for all teeth in which a permanent restoration was performed with a delay of 2 days or more. In the present study, 49 teeth were immediately restored with permanent filling after direct pulp capping. In 37 teeth, the final restoration was applied after 2–3 months. The treatment success rate was 78.4% in the group for whom Biodentine was used as a temporary filling and 85.7% in the group with immediate placement of the final restoration (the difference was not statistically significant *P* = .4031). The lack of significant differences between teeth that were restored immediately after pulp capping and teeth temporarily filled with Biodentine for 2–3 months can be explained by the relatively good physical properties of this new tricalcium silicate cement. Koubi et al. [40] determined how long Biodentine can remain as a restorative material submitted to occlusal chewing force. Koubi et al. found that this material can be used for up to 6 months. However, in our study, some restorations were discontinuous (but without the exposure of dentin) and seemed to be more yellow after 2–3 months, compared to their color directly after restoration. The discoloration of fillings may be because of their lower abrasive wear resistance, the porosity of Biodentine, and the absorption of dyes from saliva.

Some authors suggest delaying final restorations after pulp capping. According to these authors, placing the final restoration immediately after direct pulp capping complicates subsequent procedures when the teeth need root canal treatment [41]. Matsuo et al. [35] suggest that 3 months is sufficient for tentative prognoses and for determining the need for final restorations. In the aforementioned cited studies, the permanent restoration materials provided a long-term bacteria-tight seal (glass ionomer cements or resin-bonded zinc oxide eugenol cement).

## Crown discoloration

A potential drawback of the using of MTA for vital pulp therapy is the subsequent development of crown discoloration. Discoloration occurred in 60% of treated teeth when gray MTA was used as the pulpotomy material in primary teeth [42] and in 13.6% of permanent teeth after direct pulp capping with white MTA [33]. Furthermore, gray and white MTAs darken when irradiated with a curing light or fluorescent lamp in an oxygen-free environment and after contact with sodium hypochlorite or chlorhexidine gluconate [43]. The authors suggest that the bismuth oxide component of gray MTA or white MTA is responsible for the gray discoloration. In the present study, when Biodentine with zirconium oxide as radiopacifier was used for direct pulp capping, the tooth discoloration rate was 8%. In all patients, the gross observation of teeth by the naked the eye showed yellow discoloration of crowns (i.e., no gray) in comparison to the adjacent teeth. Five (83.3%) discolored teeth exhibited substantial pulp obliteration. This finding suggested that yellow discoloration of a crown is associated with dentin deposition resulting from stimulation of odontoblasts after the direct capping procedure rather than from the components of Biodentine. This is confirmed by an in vitro study in which Biodentine applied to the pulp chamber for 6 months did not induce a perceptible color change in the tooth structure [43].

## Follow-up period

The follow-up period of this study was 1–1.5 years. It is recommended that teeth should be followed after direct pulp capping for longer times to evaluate the long-term success rate. Aguilar and Linsuwanont [36] calculated the pooled success rates of 996 teeth after direct pulp capping, finding 87.5% success at 6 months to 1 year, 95.4% success at 1–2 years, 87.7% success at 2–3 years, and 72.9% success after 3 years. Similarly, Hørsted et al. [31] reported a decrease in the survival rate from 96.7% after 1 year to 81.8% after 5 years. Cho et al. [27] reported a decrease from 89.9% after 1 year to 67.4% after 3 years when MTA was used for pulp capping and from 77.7 to 52.5% when calcium hydroxide cement was used to cap exposed pulps. Furthermore, Barthel et al. [32] reported a failure rate of 44.5% after 5 years and 79.7% after 10 years, whereas Willershausen et al. [44] reported a failure rate of 19.9% after 1 year, 32.0% after 5 years, and 41.3% after 9 years.

In this study, the estimated survival rate for pulps capped with Biodentine was 82.6% after 1–1.5 years of follow-up (median 1.2 years). This result is consistent with that of Mente et al. [29], who reported a success rate of 80.5% for MTA (median follow-up 3.5 years), and the study by Hilton et al. [45], in which the success rate for direct pulp capping using MTA was 80.3% (median follow-up 1.2 years). Recently, Kundzina et al. [46] published a randomized controlled trial comparing MTA and calcium hydroxide. The success rate after direct pulp capping with MTA was 85% after 3 years of follow-up. All of the cited studies confirmed the significant superiority of MTA over calcium hydroxide. However, Schwendicke et al. [47], based on their findings and considering results from non-controlled trials, concluded that dentists can, but do not need to, use MTA instead of calcium hydroxide for direct pulp capping in permanent teeth. Thus, the 1–2 years of adequate postoperative follow-up examination, which has been applied in most studies evaluating the outcomes of direct pulp capping and our study, may well be too short. However, Jang et al. [34] suggested that most failures occur within the first 3 months.

The conventional treatment of deep carious lesions involves complete removal of all infected and affected dentin, followed by tooth restoration. However, when carious tissues are removed, the dentin barrier may be broken, making the cause of the treatment less predictable. It may also require other measures, such as direct pulp capping, partial or full pulpotomy, or in extreme cases pulpectomy [48, 49]. To minimize the potential complications of the complete removal of carious dentin close to the pulp, alternative treatment options have been proposed, such as selective excavation (the teeth will not be treated further) and stepwise excavation involving the removal of decayed tissue in two steps [49]. In deep cavitated lesions in primary or permanent teeth, selective removal to soft dentine should be performed, though stepwise removal is an option in permanent teeth [50]. Bjørndal et al. [51] found an advantage of stepwise excavation in permanent teeth, as only 17.5% of the pulps were exposed compared to 28.9% after complete excavation. Similar findings were reported by Leksell et al. [52] (18 vs. 40%) and Magnusson and Sundell [53] (15 vs. 53%). Moreover, Bjørndal et al. observed a higher success rate (74.1%) for stepwise excavation at 1 year of follow-up than direct complete excavation (62.4%) when considering exposed pulps with sustained vitality without apical radiolucency.

Aguilar and Linsuwanont [36] performed a systematic review to illustrate the clinical and radiographic success of direct pulp capping, partial pulpotomy, and full pulpotomy in vital permanent teeth with cariously exposed pulp. Overall, the success rate of each procedure was between 72.9 and 99.4%. In the present study, the overall success rate was 82.6% when Biodentine was used for direct pulp capping. However, the comparison presents difficulties, mainly due to differences in terms of case selection. For example, the patients participating in the studies were different ages (e.g., 15–44 years or 11–79 years in the present study).

The current study has some limitations. The treatment was carried out by six dentists, who mainly used saline for rinsing, sporadically sodium hypochlorite or chlorhexidine. Although all solutions are recommended to control hemorrhage [44, 54], sodium hypochlorite has been shown to inhibit the differentiation of odontoblasts from dental pulp stem cells [55] and chlorhexidine may inhibit calcium silicate cement setting [56]. In addition, the restorations were performed as either one-stage or two-stage treatments, and different adhesives and composites were used for permanent restorations. However, the quality of the temporary and final restorations recorded at 2–3 months and 1–1.5 years was acceptable in all cases.

## Conclusions

The overall success rate was 82.6% when Biodentine was used for direct pulp capping. However, a patient’s age influenced the outcomes of direct pulp capping when using this new silicate cement. Further clinical studies with longer observational time on the use of Biodentine for direct pulp capping are recommended.
